# Editorial: Urinary Incontinence in Children: Controversies Concerning the Bladder Outlet

**DOI:** 10.3389/fped.2018.00216

**Published:** 2018-08-08

**Authors:** Caroline Kuijper, Rafal Chrzan

**Affiliations:** ^1^Pediatric Urology, Emma Children's Hospital, Amsterdam UMC, University of Amsterdam, Amsterdam, Netherlands; ^2^Pediatric Urology, Jagiellonian University Medical College, Kraków, Poland

**Keywords:** urinary, incontinence, children, bladderoutlet, surgical treatment

“Becoming potty trained”: it seems so easy but for many children it is not, even when anatomy and neurologic pathways seem normal (1). An adequate assessment of the lower urinary tract is needed to make a proper diagnosis and to initiate therapy, according to the existing guidelines. Bladder dysfunction is well-defined and the treatment protocols are widely accepted, pelvic floor dysfunction can be effectively trained by the urotherapist, but still little is known about the bladder neck (BN).

The International Children's Continence Society (ICCS) has recently referred to primary BN dysfunction as a delay in it's opening but has not clearly defined it.

This research topic is focused on evaluation of the BN, looking at its anatomical substrate and finding the best diagnostic tools to interpret its function. The BN is not a static muscle but a continually sensing and reacting unit. Finally, current treatment options are discussed, both conservative and surgical.

Morphology and function of the BN can be evaluated with video urodynamic studies (VUDS), i.c. radiation. Another way to “look” at the BN is described by Schroeder et al. In their lucid article they use ultrasound (US) transperineally to evaluate the BN. They show that, in the proper atmosphere, a “normal” situation can be simulated for assessment without additional stress for the child. They give tips and tricks on how to perform this investigation and show the benefits of perineal US for the position (static-anatomical) and for the function and reaction (dynamic-functional) of the BN to coughing and holding maneuvers (2). It even provides the opportunity to train relaxation and holding maneuvers with the child while watching the US pictures and in that way is an educational and therapeutic tool.

The advantage of dynamic ultrasound is that it is a ready to use instrument in the office without radiation exposure (3, 4). However, the perineal ultrasound technique is not easy to learn, is observer dependent and in children, is only used in a few centers. Reproducibility of this method needs further evaluation.

Dobrowolska-Glazar et al. treat girls experiencing refractory urinary incontinence (UI) and signs of BN insufficiency (open and mobile BN on US and VUDS with a flat urethro-vesical angle (5)) with a known surgical technique, the Burch colposuspension. This technique creates a hammock for the “mobile” BN and in that way the “normal” detrusor-sphincter anatomical relationship and interaction can be restored. After surgery, 42% of girls were dry and 66% were UTI free and could stop antibiotic prophylaxis. The results are equivalent for both the laparoscopic and the open techniques. Although laparoscopic correction required a longer operating time, the hospital stay was shorter and the cosmetic outcome was better. Assuming that fixation of the BN is sufficient seems logical but might not be true, because the BN was also less mobile in the girls who still were incontinent. In adult women, this technique has a higher success rate. Why? Is it because the original situation was a continent one? Or does a congenital factor play a role? This topic is very controversial, emphasized in the Commentary by Podesta and González, who question the need for this type of surgery in nulliparous girls.

On the other end of the spectrum is the hypertrophied BN, in children mostly seen in boys as a result of infravesical obstruction due to urethral valves. Hennus et al. asked: what happens after incision of the hypertrophied BN in boys? Do young adults later experience retrograde ejaculation or do they have more lower urinary tract symptoms (LUTS)? Hennus et al. interviewed these patients almost 20 years after BN incision and all had antegrade ejaculation. Eight (22%) had moderate LUTS and two (5.4%) had moderate UI but this was very likely a result of the primary problem. They state that most BN hypertrophy disappears as detrusor hypertrophy diminishes. So the question rises, is the BN merely a continuum of the detrusor muscle? Although secondary BN hypertrophy is a well-known problem in boys with PUV, literature on this topic is sparse. This article demonstrates that a superficial incision does no harm on the long term but indications for and the evaluation of this procedure need more attention.

Returning to the main question: how important is the bladder neck in childhood?

Chrzan tries to clarify: *In the latest version of the standardization document of ICCS, primary bladder neck dysfunction has been mentioned for the first time. This entity is characterized by a delayed opening of the bladder neck at the beginning of the voiding phase, which is called a prolonged lag time. The lag time can be measured during invasive urodynamics and/or by means of uroflowmetry combined with electromyography (EMG) of the pelvic floor—in office* (6).

But there is also a group with a *short lag time*, that can appear as a result of a sudden opening of the pelvic floor during strong detrusor contraction provoked by the open bladder neck (7). Uroflowmetry in those patients can probably be characterized by a short flow time and a high maximal flow rate—a so-called “tower-shaped curve.”

In his article Chrzan focusses on the role of the bladder neck in female patients with refractory UI. He describes a genetic trait in women to develop UI when “it runs in the family,” but warns: “*strict criteria of the bladder neck insufficiency in children must be defined.”* Chrzan proposes diagnostic criteria for functional bladder neck insufficiency in children.

But shouldn't we use the term BN dysfunction for the whole spectrum, from insufficiency to hypertrophy?

In this research topic we have discussed the BN, which probably plays an important role in the urinary continence system but until now has been slightly neglected. We can conclude that having an anatomically normal bladder and BN and an intact sensory and reflex system does not guarantee normal function. Normalizing the position of the BN when it is too mobile and pharmacological or surgical treatment of a thickened or insufficient BN seem logical to restore the proper dimensions. But as the authors show, even a “near-normal looking” BN does not work in all patients; the BN is part of the entire continence and voiding mechanism and all components need to be taken into account.

Perhaps our view is too much BN centered: we should also look at the other pelvic organs to better understand their crosstalk. Also the influence of the brain on the bladder should not be underestimated, knowing the impact urotherapy training has on continence. So perhaps we should change our view from strictly bladder oriented to a “head-to-toe” orientation in questions involving urinary continence (8, 9).

## Author contributions

All authors listed have made a substantial, direct and intellectual contribution to the work, and approved it for publication.

### Conflict of interest statement

The authors declare that the research was conducted in the absence of any commercial or financial relationships that could be construed as a potential conflict of interest.
